# Book Review: Finding the Right Words: A Story of Literature, Grief, and the Brain

**DOI:** 10.1177/14713012231153201

**Published:** 2023-01-31

**Authors:** Sarah Howard

**Affiliations:** 5289 Manchester Metropolitan University, UK

Described as being intended as a book for families dealing with a life changing diagnosis of Alzheimer’s disease and other dementias, Cindy Weinstein has used her academic experience to build a conceptual framework to set out the complexities of her experience following her father’s diagnosis of early-onset Alzheimer’s disease with the logopenic variant in 1985, through to his passing in 1997. A chronology of the illness presents the reader at first with memories predating diagnosis (but in hindsight now leaving his daughter questioning when symptoms began), the difficulties in receiving a diagnosis, the realities of living with dementia, and the consuming anger and grief felt by everyone involved. Seamlessly, Dr Bruce Miller concludes each chapter by reflecting on the story thus far, expertly, and clearly explaining the intricacies of the brain and the reason for the changes in behaviour. He not only gives Cindy Weinstein the answers she has been looking for but also provides the reader with a thorough sound basis of understanding of the different types of dementia and how they present, the history of dementia research, and insight into current up to date findings which will be of interest to students, practitioners, people living with dementia and their caregivers. Whilst the book explains that it is not intended to be a ‘how-to’ guide for caregivers, the manner in which the scientific and medical explanations given by Dr Bruce Miller entwine with Cindy Weinstein’s own story provide a lay person with clear information, and importantly, the language, to understand what is happening to the brain behind all the medical jargon people can hear when sat in the doctor’s office.

It is clear from the onset, the title even, that this book is not only a personal narrative but a deep exploration of the far-reaching impact of dementia and grief. Written more than 30 years since the death of her father, Jerry Weinstein, his daughters words allow the audience to not only share her experience but give her father a voice and identity other than just a diagnosis of early-onset Alzheimer’s disease with the logopenic varient.

A key theme in this book is how literature, language, and words provide a place of solace and have been a means of escape and self-preservation for Cindy Weinstein, and whilst the audience may not have read the novels to which she refers, it is easy to be drawn into these different worlds right alongside her and relate the characters to her story. Retreating to this safe space of favourite literature is an example of how one can draw comfort and security when faced with the unknown and although may not be a typical routine taken by many, the ethos will be relatable to those caregivers who follow this story, as will the flood of emotions which are described so honestly and intensely. This is most poignantly seen in Chapter 2 “Word Finding” and within Cindy Weinstein’s writing under the subheading “Call Me Ahab” (Pg. 34). It is within this chapter that students and practitioners alike will be exposed to the life shifting experiences of caregivers following a dementia diagnosis and thereby learn the importance of empathy and understanding. Likewise, caregivers are likely to find comfort in the sharing of these emotions and recognise that they are not alone. Dr Bruce Miller concludes this chapter under the heading “Where Dementia Decides to Dance” (Pg. 62) by reflecting on Jerry’s experience of his first symptoms in the domain of language. Writing “where in the brain the dementia decides to dance determines the symptoms and deficits that we exhibit” (Pg. 64), he explains why people living with dementia loose those words which are in another life second nature. Although describing the clinical features of the three variants of dementia which begin the in the language regions, the reader is not overwhelmed with medical language but is instead presented with easy-to-understand examples, diagrams and tables setting out the main differences (Pg. 65). Added to these explanations is also a glossary at the end of the book, giving the reader further hands-on quick reference to knowledge when needed.

This book does not shy away from the painful realities of living with dementia or bearing witness to a loved one dying from the disease, something that hits home when Cindy herself admits that a deep denial of the situation gave her the realisation that she forgot that her father was dying. With the uncertain trajectory of any dementia diagnosis together with the relentless emotional turmoil felt by caregivers, those in a similar situation will undoubtedly relate to this scenario which is eloquently described in this book as the “very definition of denial, self-protection, and self-immolation all rolled up into one psychic hairball” (Pg. 103). Such intimate accounts of family memories allow the reader to build a picture of a father and husband and how that identity is not only lost but taken away by early-onset Alzheimer’s disease with the logopenic variant. Painful and raw memories are shared with the stigma of the disease clear to see as friends slip away quietly, unable to cope being confronted by behaviours that make them feel uncomfortable.

Cindy Weinstein’s passion for words and literature completely envelop you throughout and is only enriched by her relationship with Dr Bruce Miller. For any caregiver who has experienced a loved one receiving a diagnosis of dementia the personal journey within these pages articulates a whole host of emotions which will be familiar. For students and practitioners alike, there is an opportunity to see a person living with dementia and their family through a different lens. Presented in a format with two perspectives, that of a daughter using her love of language and literature to express her grief and that of a neurologist unpicking her story to explain the science behind her loss, this book is suitable for a wide audience including those with no medical or scientific background.

